# Racial inequities in cervical cancer mortality and the role of the Bolsa Família conditional cash transfer programme: results from the 100 Million Brazilian Cohort

**DOI:** 10.1016/j.lana.2026.101562

**Published:** 2026-07-14

**Authors:** Joanna M.N. Guimarães, Julia M. Pescarini, Ana L. Moncayo, Peter Craig, Samuel Araujo Gomes da Silva, Francine de Souza Dias, Ronal Ruiz Grijalva, Ligia Gabrielli, Isabel dos-Santos-Silva, M. da Conceição C. de Almeida, Sheila M. Alvim Matos, Ana Luísa Patrão, Gustavo Matta, Mauricio L. Barreto, Estela M.L. Aquino, Unit on the Social and Environmental Determinants of Health Inequalities (SEDHI), Pablo Alvarez, Cristiana Almeida, Cristina Elizabeth Aldaz Barreno, Roberto F.S. Andrade, Diego Oswaldo Andrade Ortiz, E.F. Anjos, Luiz Gustavo Araújo da Cruz Casais e Silva, Pamela Alejandra Arcos Garcia, Mauricio L. Barreto, R.T.I. Bernal, Mercy Julia Borbor Cordova, Elizabeth Brickley, Claire Burke, Brenda Butler, Nina Micaela Calderon Huachi, Desmond D. Campbell, Mhairi Campbell, Roberto Carrero, Laís S.M. Cardoso, Jose L. Cerezo, Irina Chis Ster, Annabel del Rocio Constatine Tigua, Philip J. Cooper, Marianne Costa, Gustavo Correa Matta, Peter Craig, Enny Paixao Cruz, Bethânia de Araujo Almeida, Edgar Marcelino de Carvalho Neto, Felix de Jesus Neves, Dandara de Oliveira Ramos, Francine de Souza Dias, Ruth Dundas, A.B.N. Einloft, Lucas Emanuel da Silva, A.A. Fonseca, Roberto Fernandes Silva Andrade, Rosemeire Fiaccone Leovigildo, María del Pilar Flores-Quispe, Ligia Franco Sansigolo Kerr, Gervásio Ferreira dos Santos, Emmanuelle Goes, Marcos Gonzaga, Monsermin Gualan, J.M.N. Guimarães, Sally Hargreaves, Katie Harron, Évelin Angélica Herculano de Morais, Maria Yury Ichihara, Vittal Katikireddi, Carl Kendall, A.H. Leyland, R.J. Lilford, Luz Marina Llangari Arizo, David Lopes, Rachel Lowe, Daniela Lugo, Sara Macdonald, D.C. Malta, Adelyne Mendes Pereira, Adriana Mendoza Ruiz, Ana Lucía Moncayo Benalcazar, Denise Moraes Pimenta, Grace Navarrete Chavez, Erica Miranda Nascimento, Rumão Batista Nunes de Carvalho, Sara Caroline Oliveira Junker, Samila Oliveira Lima Sena, Jonathan R. Olsen, Suelen Oliveira, Naiá Ortelan, E.P. Pinto Junior, Robespierre Pita, Julia M. Pescarini, Rita Ribeiro, Rodrigo Alejandro Rodriguez Alvarado, Batul Rojeab Bravo, Natalia Cristina Romero Sandoval, Fernanda Revoredo, Maria Alejandra Ruano, Nai Rui Chng, Ronal Ruiz, Raphael Sande, T. Santos de Jesus, Paula M. Santucci, Katie Ellen Scandrett, Mariana Sebastiao, Viviane Silva de Jesus, Michal Shimonovich, Maira Lima Souza, J.F. Sousa Filho, Raiza Tourinho Lima, Cleônidas Tavares de Souza Junior, Carlos Teles, David Gonzalo Vera Alcivar, Cristiani Vieira Machado, Valerie Wells, Daniel Zurita

**Affiliations:** a Center for Data and Knowledge Integration for Health (CIDACS) – Fiocruz, Salvador, Brazil; b Faculty of Epidemiology and Population Health – London School of Hygiene & Tropical Medicine, London, UK; c Centro de Investigación para la Salud en América Latina (CISeAL), Pontificia Universidad Católica del Ecuador, Quito, Ecuador; d Health Economics and Health Technology Assessment, School of Health and Wellbeing, University of Glasgow, Glasgow, UK; e Universidad Internacional del Ecuador, Quito, Ecuador; f Instituto de Saúde Coletiva – Universidade Federal da Bahia, Salvador, Brazil; g Gonçalo Muniz Institute – Fiocruz, Salvador, Brazil; h Faculty of Psychology and Education Science, Center for Psychology, University of Porto, Porto, Portugal

**Keywords:** Cervical cancer mortality, Racial inequities, Bolsa Família Programme, Conditional cash transfer, Brazil, 100 Million Brazilian Cohort, Social determinants of health, Health equity

## Abstract

**Background:**

Structural and institutional racism in Brazil mainly affects Black, ‘Parda’(Brown/Mixed) and Indigenous women, who are less likely to receive adequate health care. We investigated the association between race and cervical cancer mortality and whether this association is attenuated by receipt of the Bolsa Família Programme (BFP).

**Methods:**

Women from the 100 Million Brazilian Cohort were linked to nationwide mortality registries (2004–2015). Self-reported race (White/Black/Parda/Asian/Indigenous women) and cervical cancer mortality (ICD10C53) were analysed using Poisson models adjusted for age, education, area of residence (rural/urban), and year of enrolment. Effect modification by BFP (yes/no) was assessed.

**Findings:**

We analysed 18,291,600 women. Cervical cancer mortality rates were greater among Indigenous (adjusted-Mortality rate ratio (aMRR) and adjusted-Mortality rate difference (aMRD): 1.78, 95% CI 1.40–2.25 and 5.26, 95% CI 2.41–8.11, respectively), Asian (aMRR = 1.55, 1.16–2.06 and aMRD = 3.72, 0.74–6.70), Parda (aMRR = 1.28, 1.22–1.34 and aMRD = 1.91, 1.56–2.25), and Black (aMRR = 1.20, 1.12–1.29 and aMRD = 1.37, 0.80–1.95) women vs. White women. Among BFP recipients, aMRR and aMRD were, respectively, 1.12 (95% CI 1.03–1.22) and 0.77 (95% CI 0.19–1.35) for Black, 1.25 (1.19–1.32) and 1.61 (1.25–1.97) for Parda, 1.74 (1.35–2.24) and 4.70 (1.94–7.47) for Indigenous, and 1.56 (1.12–2.18) and 3.57 (0.26–6.87) for Asian women vs. White women; whilst among non-BFP recipients, aMRR and aMRD were, respectively, 1.53 (95% CI 1.31–1.78) and 4.05 (95% CI 2.34–5.76) for Black, 1.36 (1.24–1.49) and 2.76 (1.94–3.59) for Parda, 1.98 (0.94–4.16) and 7.48 (−3.77 to 18.73) for Indigenous, and 1.55 (0.89–2.68) and 4.21 (−2.27 to 10.68) for Asian women vs. White women (p interaction = 0.017).

**Interpretation:**

Racial differentials in cervical cancer mortality were reduced among BFP recipients and heightened among non-BFP recipients. This suggests that racial inequities in cervical cancer mortality may be mitigated with BFP receipt, through cash and/or conditionalities. Possibly, by improving women’s income and access to cervical cancer screening and pre-cancer treatment, ultimately reducing mortality.

**Funding:**

National Institute for Health and Care Research-NIHR, Wellcome Trust, CNPq.

## Resumo

**Introdução:**

O racismo estrutural e institucional no Brasil afeta principalmente mulheres Pretas, Pardas e Indígenas, que têm menor probabilidade de receber cuidados de saúde adequados. Investigamos a associação entre raça e mortalidade por cancer de colo de útero e se essa associação é atenuada pelo recebimento do Programa Bolsa Família (PBF).

**Método:**

As mulheres da Coorte de 100 Milhões de Brasileiros foram vinculadas aos registros nacionais de mortalidade (2004–2015). A raça/cor autorreferida (mulheres Brancas/Pretas/Pardas/Amarelas/Indígenas) e a mortalidade por cancer de colo de útero (CID10C53) foram analisadas utilizando modelos de Poisson ajustados por idade, escolaridade, área de residência (rural/urbana) e ano de entrada na coorte. Avaliou-se a modificação de efeito pelo PBF (sim/não).

**Resultados:**

Foram analisadas 18.291.600 mulheres. As taxas de mortalidade por cancer de colo de útero foram maiores entre as mulheres Indígenas (Razão da taxa de mortalidade ajustada (RTMa) e Diferença da taxa de mortalidade ajustada (DTMa): 1.78, IC 95% 1.40–2.25 e 5.26, IC 95% 2.41–8.11, respectivamente), Amarelas (RTMa = 1.55, 1.16–2.06 e DTMa = 3.72, 0.74–6.70), Pardas (RTMa = 1.28, 1.22–1.34 e DTMa = 1.91, 1.56–2.25) e Pretas (RTMa = 1.20, 1.12–1.29 e DTMa = 1.37, 0.80–1.95) vs. mulheres Brancas. Entre as beneficiárias do PBF, as RTMa e DTMa foram, respectivamente, 1.12 (IC 95% 1.03–1.22) e 0.77 (IC 95% 0.19–1.35) para mulheres Pretas, 1.25 (1.19–1.32) e 1.61 (1.25–1.97) para Pardas, 1.74 (1.35–2.24) e 4.70 (1.94–7.47) para Indígenas, e 1.56 (1.12–2.18) e 3.57 (0.26–6.87) para Amarelas vs. mulheres Brancas; enquanto entre as não-beneficiárias do PBF, as RTMa e DTMa foram, respectivamente, 1.53 (IC 95% 1.31–1.78) e 4.05 (IC 95% 2.34–5.76) para mulheres Pretas, 1.36 (1.24–1.49) e 2.76 (1.94–3.59) para Pardas, 1.98 (0.94–4.16) e 7.48 (−3.77 to 18.73) para Indígenas, e 1.55 (0.89–2.68) e 4.21 (−2.27 to 10.68) para Amarelas vs. mulheres Brancas (p-valor interação = 0.017).

**Interpretação:**

Os diferenciais raciais na mortalidade por cancer de colo de útero foram reduzidos entre as beneficiárias do PBF e aumentados entre as não-beneficiárias do programa. Isso sugere que as desigualdades raciais na mortalidade por cancer de colo de útero podem ser mitigadas pelo PBF, através das transferências de renda e/ou das condicionalidades. Possivelmente, devido ao aumento da renda e do acesso das mulheres ao rastreamento do cancer de colo de útero e tratamento pré-cancerígeno, reduzindo a mortalidade.

**Research in context**

**Evidence before this study**

We searched PubMed to August 3, 2025 with a title and abstract restriction using the following terms: (“race” OR “racism” OR “ethnicity” OR “ethnoracial”) AND (“cervical cancer”) AND (“mortality” OR “death” OR “survival”), which returned 398 articles published since 1986. When we repeated the same search by adding the terms: AND (“cash transfer” OR “social protection” OR “poverty-alleviation” OR “bolsa família”), no study was found. From those 398 publications, 103 have assessed race differences on cervical cancer (CC) mortality, but only a few have examined racial inequities in CC mortality. Many of them have evaluated samples of CC patients and investigated race as a prognostic factor or a biological trait, without addressing racial inequities and their structural drivers, including institutional racism. Overall, the studies reported higher CC mortality and poorer survival for Black women and better outcomes for White women. Indigenous peoples were rarely included in studies, although they often present the highest CC mortality rates. Around 85% of studies are from the United States (n = 88) and the remaining from the UK (n = 1), Canada (n = 1), New Zealand (n = 1), South Africa (n = 3), and Brazil (n = 9). Brazilian studies have used ecological designs, were limited to specific geographical areas, and have not empirically addressed the potential of strategies to reduce racial inequities in CC mortality.

**Added value of this study**

This comprehensive study using individual-level nationwide data for over 20 million women, showed striking racial inequities in CC mortality rates, with Indigenous, Black and Parda women having the highest CC mortality rates vs. White women. Unlike previous research, our study demonstrated an interaction between race and receipt of the Bolsa Família Programme (BFP), the world’s largest conditional cash transfer programme, such that racial differentials in CC mortality were reduced among BFP recipients, and heightened among BFP non-recipients. Our study is the first to investigate the potential of a conditional cash transfer programme (the BFP) in reducing racial inequities in CC-related outcomes.

**Implications of all the available evidence**

Conditional cash transfers impact women’s income and empowerment, providing them with resources to afford food, medications, and transportation to healthcare settings. Also, programme’s conditionalities promote greater access to education and primary healthcare, facilitating HPV vaccination and CC screening. Therefore, BFP might help to reduce socioeconomic inequalities and barriers of access to healthcare for Black, Parda and Indigenous women relative to White women, reducing racial inequities in CC mortality. As policy implications and external public health recommendations, we suggest affirmative actions to ensure a more racially diverse healthcare workforce to reduce racial bias in healthcare and institutional racism. We also suggest the inclusion of HPV vaccination and CC screening (e.g., Pap testing) to BFP conditionalities, with special attention to Indigenous, Black and Parda women.

## Introduction

Cervical cancer (CC) is the fourth most common cancer in women in incidence and mortality globally,^1^^,^^2^ with substantial differences across and within countries.^1^, ^2^, ^3^ The highest rates of CC mortality occur in low- and middle-income countries as a result of major inequities in the access to HPV (human papilloma virus) vaccination, CC screening (Pap tests), diagnosis and treatment services, driven by social determinants such as poverty, poorer education and lower levels of human development.^1^, ^2^, ^3^ In Brazil, a middle-income country marked by historical and current social and racial inequalities, CC is still the third most common cancer in women in incidence and mortality,^4^ and disproportionately affects the poorest people and marginalized racial groups.^5^^,^^6^ The World Health Organization (WHO) has set three goals to eliminate CC as a public health problem by 2030: HPV complete vaccination in 90% of girls, screening 70% of women, and treating 90% of women with cervical disease.^7^ Despite this, in 2019 in Brazil, complete HPV vaccination coverage for girls was only 47.4%.^8^ Although it is a preventable and curable disease if diagnosed early and promptly treated,^3^ evidence from high-income countries shows CC inequities by race in HPV vaccination,^9^ screening,^10^ incidence,^11^ survival^12^ and mortality,^12^ with poorer outcomes for Black women.

Structural and institutional racism in Brazil mainly affects Black, ‘Parda’(Brown/Mixed) and Indigenous women, who are systematically less likely to receive adequate health care compared to White women.^13^^,^^14^ Black, Parda and Indigenous Brazilian women are subject to multiple expressions of discrimination (e.g., racism and sexism), reducing their opportunities for education, income and employment over the lifecourse.^13^ In turn, this leads them to be uninsured and live in households with poor conditions and in segregated areas with fewer health services and of poorer quality, with transportation and security related issues.^13^, ^14^, ^15^ These social and structural barriers impact access to reproductive health services, gynaecological appointments and screening tests for CC, leading to late diagnosis in advanced stages and increased CC mortality,^5^^,^^6^^,^^16^ despite the free and universal health-care system in Brazil. Although some studies from Brazil^5^^,^^6^^,^^17^ and other Latin American countries^18^ showed CC mortality to be highest among Indigenous and Black women, many Latin American surveys and mortality registries do not collect data on race.^18^^,^^19^ Moreover, many Brazilian researchers tend to exclude Indigenous people from health analyses, reflecting their historical trajectory of discrimination and invisibility linked to structural racism.^19^^,^^20^

Brazil’s Bolsa Família Programme (BFP) was implemented in 2004 and is the world’s largest conditional cash transfer programme,^21^^,^^22^ targeted at low and extremely low-income families. The BFP aims to i. alleviate poverty by providing a minimum level of income for families, and ii. break the intergenerational transmission of poverty by requiring beneficiaries to comply with conditionalities (minimum school attendance and vaccination for children and adolescents, and pre- and post-natal visits for women).^23^ The BFP has been able not only to reduce poverty and income inequalities, but also to reduce health inequalities, through acting on social determinants (e.g., improved access to education, food and health services).^21^, ^22^, ^23^ Previous studies have reported positive impacts of the BFP on child^21^ and maternal mortality,^24^ cardiovascular diseases,^22^ mental health outcomes,^25^ breast cancer mortality,^26^ and others. Conditional cash transfers have been effective in increasing the uptake of health care services, CC screening tests and promoting safe sexual behaviors in Latin American countries.^27^^,^^28^ However, no study has investigated the relationship between cash transfer programmes and CC-related mortality.

Previous research on racial disparities in CC mortality in Brazil have used ecological study designs, were limited to specific Brazilian states or municipalities, and have not empirically addressed the potential of strategies to reduce racial differentials in CC mortality. Using individual-level nationwide data from a large-scale administrative cohort linked to mortality data,^23^ the aims of this study were to investigate the association between race and CC mortality and assess whether this is modified by being a BFP recipient. We hypothesise that Black, Parda, and Indigenous women have a higher mortality rate from CC than White women, and that these racial inequities could be reduced among BFP recipients.

## Methods

### Study design and participants

We conducted a longitudinal study within the 100 Million Brazilian Cohort (100MCohort). The 100MCohort is a nationwide administrative cohort assembled from the Brazilian Government’s Unified Register for Social Programmes (CadUnico), which includes data from over 114 million low-income Brazilians (nearly 55% of the country’s population) for the 2001–2015 period.^23^ To be registered in CadUnico, families are required to apply and have an income ≤1/2 minimum wage per capita (approximately $125 USD in 2020) or a total family income ≤3 times the minimum wage (approximately $750 USD).^23^ For this study, the 100MCohort baseline dataset was linked to the Brazilian Mortality Database to identify women in the cohort who had died from CC during follow-up. Detailed linkage procedures can be found elsewhere.^23^ The study was approved by the Ethics Committee of Instituto Gonçalo Moniz—Oswaldo Cruz Foundation (CAAE 77084624.5.0000.0040), considering the study’s ethical aspects on risk-benefit analysis, participants’ rights, measures to prevent harm to and discrimination of individuals or groups and researcher responsibilities. Since data was obtained from secondary datasets, participants’ informed consent was waived.

### Measures

Exposure—Race/skin color was used as a social and historical (not a biological) construct, i.e., a marker of the perceived effect of life experiences of racism.^5^ Data on self-reported race/skin color were collected at the time of enrolment in CadUnico (cohort baseline) via face-to-face interviews using the classification officially adopted by the Brazilian Census (IBGE): White, ‘Parda’ (or Brown/Mixed, a proxy for people of Black and White race/skin color), Black, ‘Yellow’ (Asian or people of Asian descent), or Indigenous (Brazilian Indigenous) people.

Outcome—Individual information on the occurrence of deaths during follow-up, including the dates and their underlying cause coded according to the International Classification of Diseases (ICD-10), was obtained through linkage to the National Mortality Database. Deaths from CC were those with an underlying cause coded as C53, according to ICD-10.

Effect modifier—To be eligible for BFP, families must be registered in CadUnico and be low or extremely low-income.^22^^,^^23^ The BFP status was defined as whether the participant was a recipient (yes/no) after enrolment in CadUnico and before dying. BFP recipients were those who were paid the benefit (preferably a woman, who received it on behalf of the family) or were members of a payee’s household. We assumed that when individuals started receiving BFP they continued receiving it for the remaining study period, since only 0.2% of BFP recipients in our cohort stopped receiving before the end of follow-up.

Covariates—Data on covariates were also collected at enrolment into CadUnico, including individual information on age (continuous), education level (categorized as ≤5, 6–9, >9 years of schooling), area of residence (rural vs. urban), and women’s year of enrolment into the cohort.

### Statistical analysis

**Fig. 1:**
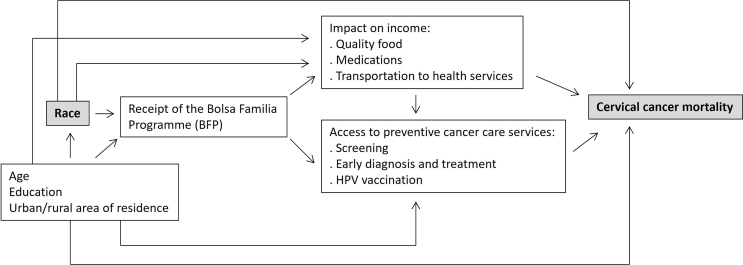
Conceptual model for the association of race with cervical cancer mortality and effect modification by Bolsa Família Programme (BFP).

The sample characteristics were described across race categories, and differences were tested using chi-square tests and analysis of variance (sample characteristics were also described across BFP receipt groups (yes/no)). Time at risk was calculated from the time a woman was enroled into CadUnico to the time of her death from CC, death from another cause, or the end of follow-up (for this analysis, December 31, 2015), whichever occurred first. Since we are modeling count data and analysing mortality rates (number of CC deaths per woman-years at risk), Poisson regression models were used to estimate adjusted-Mortality rate ratios (aMRR) and 95% confidence intervals (95% CI). Associations of race with CC mortality were estimated after adjustments for women’s age only (Model 1) and simultaneously for age and education (Model 2), to account for age and education differences by race categories. Then, we added to Model 3 the area of residence (rural vs. urban) to account for socioeconomic differences of municipalities where women with different self-reports of race lived, and the year of women’s enrolment in the cohort, to determine racial differentials after accounting for differences in the follow-up period by race. Model 4 added the BFP recipient variable to estimate the association of being a recipient of the BFP with CC mortality (Conceptual model, Fig. 1). We also estimated adjusted-Mortality rate differences (aMRD) and 95% CI using marginal predicted means from the fitted Poisson models.

To investigate whether BFP receipt modified the association between race and CC mortality, a multiplicative interaction term between race and BFP receipt was added to Model 4. If the interaction term was significant (p < 0.05) based on likelihood ratio test, stratified effects were obtained by calculating the aMRR and aMRD for race within strata of BFP receipt.

We conducted two sensitivity analyses. First, we restricted the analysis to Brazilian municipalities known to have high proportion of death registration (≥95%), to assess the extent to which the observed associations of race and mortality might reflect the poorer quality of the data (i.e., underreporting of mortality among the poorest). Second, we restricted the sample to low-educated women (those who never went to school or attended ≤5 years of schooling), who were more likely to be eligible for BFP (i.e., women from low or extremely low-income families), to check whether our associations hold.

We have also performed a couple of additional analyses. Firstly, we have added the Brazilian region of residence to the fully adjusted model, to account for potential bias due to differences in the quality of the mortality database across regions. Secondly, although our study hypotheses were on racial health inequities and how they are modified by BFP, we have also estimated associations between BFP and CC mortality, stratified by race. Thirdly, we checked for the overdispersion of the Poisson regression model (i.e., when the variance of the count of events is greater than its mean). All statistical analyses were performed in Stata, version 15.1.

### Role of the funding source

The study’s funders had no role in the study design; in the collection, analysis, and interpretation of data; in the writing of the report; and in the decision to submit the manuscript for publication.

## Results

**Fig. 2:**
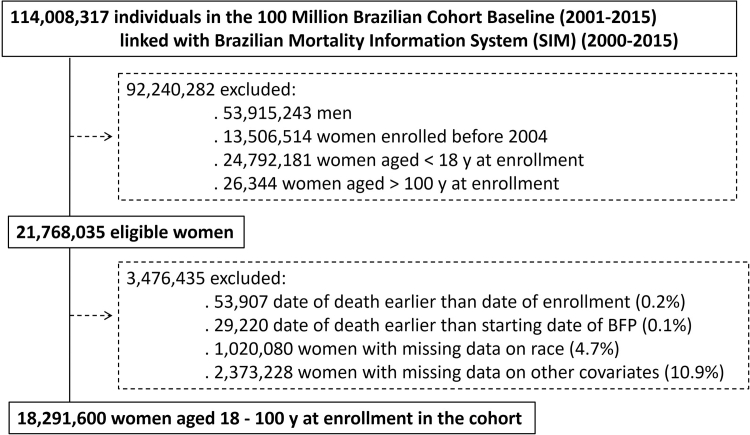
Flowchart of participants’ selection.

All women enroled in CadUnico between January 1, 2004 (because levels of data missingness were high for those enroled in previous years) and December 31, 2015 (the last year for which mortality data were available), and who were aged between 18 and 100 years at enrolment, were eligible to participate in this study. Women whose date of death was earlier than their date of enrolment into the cohort, or earlier than the starting date of BFP receipt (probably due to linkage errors) were excluded. Women with missing data on race (4.7%, n = 1,020,080) or other socioeconomic covariates (10.9%, n = 2,373,228) were also excluded, leaving 18,291,600 women for analysis (Fig. 2).

Among the 18,291,600 studied women, the number of deaths by CC was 9573 (0.05%) (White: 0.04%, n = 2817; Parda: 0.05%, n = 5673; Black: 0.06%, n = 965; Asian: 0.05%, n = 48; Indigenous women: 0.07%, n = 70). BFP recipients and non-recipients were 13,026,278 (71.2%) and 5,265,322 (28.8%), respectively. Indigenous, Black, and Parda women were more likely to be BFP recipients, younger, and less educated (Table 1). When comparing women who received and did not receive BFP, non-recipients were more likely to be White women, older, higher educated, and tended to live in more urban municipalities, than BFP recipients (Supplementary Table S1).

**Table 1:** Characteristics of the study population, by self-reported race. 100 Million Brazilian Cohort (2004–2015), N = 18,291,600 women aged 18–100 years.

Variables	Overall	Self-declared race groups					
		White	Parda (Brown/mixed)	Black	Asian	Indigenous	p-value
	n = 18,291,600	n = 6,333,911	n = 10,189,340	n = 1,581,579	n = 88,469	n = 98,301	
Self-declared race, %	–	34.6	55.7	8.6	0.5	0.5	–
Cervical cancer deaths, n (%)	9573 (0.05)	2817 (0.04)	5673 (0.05)	965 (0.06)	48 (0.05)	70 (0.07)	<0.0001
Bolsa Família recipient, n (%)							
Yes	13,026,278 (71.2)	3,984,316 (62.9)	7,679,654 (75.4)	1,218,409 (77.0)	54,741 (61.9)	89,158 (90.7)	<0.0001
No	5,265,322 (28.8)	2,349,595 (37.1)	2,509,686 (24.6)	363,170 (23.0)	33,728 (38.1)	9143 (9.3)	
Age in years at baseline, mean (SD)	37.1 (15.8)	39.0 (16.5)	35.9 (15.3)	37.3 (15.4)	38.0 (16.4)	32.6 (14.0)	<0.0001
Education in years, n (%)							
>9	5,081,785 (27.8)	1,883,835 (29.7)	2,771,254 (27.2)	380,957 (24.1)	31,261 (35.3)	14,478 (14.7)	<0.0001
6–9	5,150,342 (28.2)	1,770,849 (28.0)	2,885,078 (28.3)	451,138 (28.5)	21,336 (24.1)	21,941 (22.3)	
≤5	8,059,473 (44.1)	2,679,227 (42.3)	4,533,008 (44.5)	749,484 (47.4)	35,872 (40.5)	61,882 (62.9)	
Area of residence, n (%)							
Urban	14,582,160 (79.7)	5,295,723 (83.6)	7,874,491 (77.3)	1,308,043 (82.7)	71,259 (80.6)	32,644 (33.2)	<0.0001
Rural	3,709,440 (20.3)	1,038,188 (16.4)	2,314,849 (22.7)	273,536 (17.3)	17,210 (19.4)	65,657 (66.8)	

Age-standardized CC mortality rates in the overall cohort were higher for Indigenous women (19.3/100,000 woman-years), Asian (16.8/100,000), Parda (14.6/100,000), Black (14.2/100,000), and lowest for White women (10.7/100,000). When looking into BFP recipient groups (yes/no), a similar pattern in mortality rates by race was seen for both BFP groups. However, non-BFP recipient women showed slightly lower mortality rates, compared to BFP recipient women (Supplementary Fig. S1).

After full adjustments for age, education, area of residence, year of enrolment and BFP recipient, CC mortality rates were greater among Indigenous (aMRR = 1.78, 95% CI 1.40–2.25), Asian (aMRR = 1.55, 1.16–2.06), Parda (aMRR = 1.28, 1.22–1.34) and Black (aMRR = 1.20, 1.12–1.29) women vs. White women (Model 4). After full adjustments, CC mortality rates were lower among women not receiving BFP than BFP recipients (aMRR = 0.68, 0.64–0.72) (Model 4) (Table 2).

**Table 2:** Association of race with cervical cancer mortality. 100 Million Brazilian Cohort (2004–2015), N = 18,291,600 women aged 18–100 years.

	aMRR (95% CI)			
	Model 1	Model 2	Model 3	Model 4
Race				
Parda (Brown/mixed) vs. White	1.33 (1.27,1.39)^a^	1.30 (1.24,1.36)^a^	1.31 (1.25,1.37)^a^	1.28 (1.22,1.34)^a^
Black vs. White	1.31 (1.21,1.41)^a^	1.26 (1.17,1.36)^a^	1.24 (1.15,1.33)^a^	1.20 (1.12,1.29)^a^
Asian vs. White	1.48 (1.11,1.97)^b^	1.50 (1.13,1.99)^c^	1.56 (1.17,2.08)^d^	1.55 (1.16,2.06)^e^
Indigenous vs. White	1.78 (1.41,2.26)^a^	1.62 (1.27,2.05)^a^	1.87 (1.48,2.38)^a^	1.78 (1.40,2.25)^a^
Age at baseline per 5-year increase	1.04 (1.04,1.04)^a^	1.04 (1.04,1.04)^a^	1.04 (1.04,1.04)^a^	1.04 (1.04,1.04)^a^
Education				
6–9 years vs. >9 years	–	1.90 (1.74,2.07)^a^	1.77 (1.62,1.93)^a^	1.74 (1.59,1.89)^a^
≤5 years vs. >9 years	–	2.46 (2.27,2.67)^a^	2.35 (2.16,2.56)^a^	2.29 (2.10,2.49)^a^
Area of residence		–		
Rural vs. Urban	–	–	0.70 (0.66,0.74)^a^	0.70 (0.66,0.74)^a^
Year of enrolment per 1-year increase	–	–	0.95 (0.95,0.96)^a^	0.97 (0.96,0.98)^a^
Bolsa Família Programme recipient				
No vs. Yes	–	–	–	0.68 (0.64,0.72)^a^

Note: aMRR, adjusted-Mortality rate ratio; CI, Confidence interval.

**Model 1**: + Age.

**Model 2**: Model 1 + Education.

**Model 3**: Model 2 + Area of residence + Year of enrolment.

**Model 4**: Model 3 + Bolsa Família Programme (BFP) recipient.

a p-value<0.0001.

b p-value = 0.007.

c p-value = 0.005.

d p-value = 0.002.

e p-value = 0.003.

Adjusted mortality rate differences (aMRD) indicated that Indigenous women had an excess rate of 5.26 additional deaths (per 100,000 woman-years) compared to White women (aMRD = 5.26, 95% CI 2.41–8.11). Asian (aMRD = 3.72, 0.74–6.70), Parda (aMRD = 1.91, 1.56–2.25) and Black (aMRD = 1.37, 0.80–1.95) women also showed increased rates vs. White women (not shown in table).

**Fig. 3:**
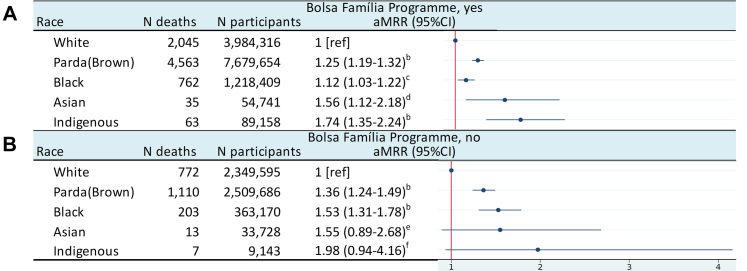
Association of race with cervical cancer mortality, stratified by Bolsa Familia Programme (BFP) recipient. A = Bolsa Família Programme, yes. B = Bolsa Família Programme, no. ^a^100 Million Brazilian Cohort (2004–2015), N = 18,291,600 women aged 18–100 years. **Note:** aMRR, adjusted-Mortality rate ratio. CI, Confidence interval. BFP, Bolsa Família Programme. ^a^Adjusted for age + education + area of residence (Rural/urban) + year of enrolment. p for interaction = 0.017. ^b^p-value < 0.0001, ^c^p-value = 0.007, ^d^p-value = 0.009, ^e^p-value = 0.12, ^f^p-value = 0.07.

There was evidence of a multiplicative interaction between race and BFP recipient (p interaction = 0.017), indicating that the relationship between race and CC mortality was different for women who received and those who did not receive BFP. Among BFP recipients strata, Black, Parda and Indigenous women vs. White women had a 12%, 25% and 74% greater mortality rate from CC (aMRR = 1.12, 1.03–1.22 (p = 0.007); aMRR = 1.25, 1.19–1.32 (p < 0.0001); and aMRR = 1.74, 1.35–2.24 (p < 0.0001), respectively), whilst among non-BFP recipients strata, Black, Parda and Indigenous women vs. White women had a 53%, 36% and 98% higher mortality rate from CC (aMRR = 1.53, 1.31–1.78 (p < 0.0001); aMRR = 1.36, 1.24–1.49 (p < 0.0001); and aMRR = 1.98, 0.94–4.16 (p = 0.07), respectively). Asian vs. White women had a 56% and 55% higher mortality rate among BFP recipients (aMRR = 1.56, 1.12–2.18 (p = 0.009)) and non-recipients (aMRR = 1.55, 0.89–2.68 (p = 0.12)), respectively. Estimates for non-BFP recipient Indigenous and Asian women were based on smaller numbers and wider CIs that crossed 1.0, however (Fig. 3).

Among BFP recipients, adjusted mortality rate differences were 0.77, 1.61, 4.70 and 3.57 for Black (aMRD = 0.77, 0.19–1.35 (p = 0.009)), Parda (aMRD = 1.61, 1.25–1.97 (p < 0.0001)), Indigenous (aMRD = 4.70, 1.94–7.47 (p = 0.001)) and Asian women (aMRD = 3.57, 0.26–6.87 (p = 0.034)) vs. White women. Among non-BFP recipients, adjusted mortalitity rate differences were 4.05, 2.76, 7.48 and 4.21 for Black (aMRD = 4.05, 2.34–5.76 (p < 0.0001)), Parda (aMRD = 2.76, 1.94–3.59 (p < 0.0001)), Indigenous (aMRD = 7.48, −3.77 to 18.73 (p = 0.19)) and Asian (aMRD = 4.21, −2.27 to 10.68 (p = 0.20)) vs. White women (not shown in table).

Sensitivity analysis restricted to Brazilian municipalities with high proportion of death registration (≥95%) yielded similar overall findings, although aMRR slightly increased for Indigenous vs. White women (from 1.78, 1.40–2.25 (p < 0.0001) to 2.05, 1.33–3.16 (p = 0.001)) and slightly decreased and was no longer significant for Asian women (from 1.55, 1.16–2.06 (p = 0.003) to 1.34, 0.78–2.31 (p = 0.29)) (Supplementary Table S2). A second sensitivity analysis restricting the sample to low-educated women (more likely to be low or extremely low-income women and therefore more prone to be eligible for BFP) showed virtually the same results with a slightly increase in aMRRs for all race groups, although for Asian women it decreased and was no longer significant (Supplementary Table S3).

Additional analysis further adjusted for the Brazilian region of residence yielded similar associations between race and CC mortality, albeit the MRR for Indigenous vs. White women decreased from 1.78 (95% CI 1.40–2.26) to 1.46 (1.15–1.86) (Supplementary Table S4). Race-specific models for the association between BFP and CC mortality showed that mortality rates were lower for BFP non-recipient vs. BFP recipient women. However, MRRs were significant only among strata of White and Parda women (aMRR = 0.68, 95% CI 0.61–0.75, and aMRR = 0.66, 0.61–0.71, respectively) (Supplementary Table S5). The evaluation of overdispersion of the Poisson model indicated that the variance in the data was greater than the mean. A quasi-Poisson model was then estimated, with very similar results (Supplementary Table S6).

## Discussion

This comprehensive study, using individual-level longitudinal data for over 20 million women, showed striking racial inequities in CC mortality rates, with Indigenous women having the highest CC mortality rates vs. White women. We also showed an interaction between race and BFP, such that racial differentials in CC mortality for Black, Parda and Indigenous women were reduced among BFP recipients, and heightened among BFP non-recipients. For example, Black vs. White women who did not receive BFP had a 53% (aMRR = 1.53, 1.31–1.78) higher mortality rate and an excess mortality rate of 4.05 (aMRD = 4.05, 2.34–5.76), whilst Black vs. White women who received BFP had a 12% (aMRR = 1.12, 1.03–1.22) higher mortality rate and an excess mortality rate of 0.77 (aMRD = 0.77, 0.19–1.35), with no overlapping 95% CIs. To our knowledge, this is the first study to investigate the potential of a conditional cash transfer programme (the BFP) in reducing racial inequities in CC-related outcomes.

Indigenous women have a heightened burden of CC, due to a complex interplay of low socioeconomic status, geographical isolation and communication barriers with health care providers.^17^^,^^18^ Additionally, cultural factors such as early sexual exposure, multiparity and multiple sexual partners lead to a high prevalence of HPV infection (e.g., 45.9% in Yanomami women),^29^ coupled with a low receipt of preventive Pap tests and vaccines.^17^^,^^18^^,^^29^ Our study also showed that Black and Parda women had, respectively, 20% and 28% higher CC mortality rates than their White counterparts. Consistent with our results, an ecological study from all Brazilian municipalities between 2000 and 2020 showed that CC mortality rates were higher in Black women (RR = 1.27) and dramatically higher in Indigenous women (RR = 1.82), compared to White women.^17^

Due to institutional racism and racial bias in health care, Black and Parda women from Brazil,^16^ and African-american women from the US,^10^, ^11^, ^12^ have significantly fewer Pap tests, and more late CC diagnoses at advantaged stages with worse prognosis, increasing their CC mortality rates. Analysis of nationwide ecological data from hospital-based cancer registries (Registros Hospitalares de Cancer-RHC) in Brazil (2000–2012) showed that Indigenous and Black women presented higher odds of having late (III or IV) stage CC at diagnosis (OR = 2.38, 95% CI 1.06–5.33 and OR = 1.16, 95% CI 1.02–1.31, respectively) vs. White women.^16^

In our study, Asian women presented a 55% greater CC mortality rate relative to White women. However, this result should be interpreted with caution because self-reported Asian race/skin color in Brazil (in Portuguese, *Amarela* or Yellow) is subject to misclassification, especially among the poorest. For example, some states from Northern (poorer) Brazil have a larger self-reported ‘Yellow’ population than states from the Southeast (wealthier) region like São Paulo, despite the latter has the country’s largest historical concentration of Japanese immigrants.^30^ Although the category ‘yellow’ was defined in the Brazilian Census to represent Asian migrants and their descendents, the nonspecific nature of this category can lead to data inconsistencies, e.g., the possible overlap with the Parda category.^30^ Indeed, in the sensitivity analysis restricted to municipalities with better quality mortality data, aMRR decreased and was no longer significant for Asian women.

We showed that among BFP recipients, racial inequities in CC mortality were reduced, such that for Black, Parda and Indigenous women vs. White women, the association between race and mortality was attenuated among recipients. Conditional cash transfers impact women’s income and empowerment, providing them with resources to afford food, medications, and transportation to health care settings.^21^^,^^22^ Also, programme’s conditionalities promote greater access to education and primary health care, facilitating HPV vaccination and CC screening.^28^ Therefore, BFP might help to reduce socioeconomic inequalities and barriers of access to health care for Black, Parda and Indigenous women relative to White women, reducing racial inequities in CC mortality. A randomized study of PROGRESA (a conditional cash transfer programme in Mexico, on which CC screening is part of the programme’s conditionalities) found spillover effects in the demand for CC screening among ineligible women, possibly due to social interactions with eligible households.^31^ In Brazil, BFP was associated with increased access to Pap tests (PR = 1.26, 95% CI 1.13–1.40) and guidance on CC screening (PR = 1.13, 95% CI 1.07–1.19).^27^ Moreover, a recent systematic review showed positive impacts of cash transfer programmes in promoting safe sexual behaviors such as delayed sexual debut, marriage and first pregnancy among girls, increased condom use, and decreased multiple partners, reducing HPV infection risk.^28^

Despite this evidence showing protective effects of cash transfer programmes on CC-related outcomes, our results indicated that BFP non-recipient women had lower CC mortality rates than BFP recipients (aMRR = 0.68, 95% CI 0.64–0.72), in contrast to our expectations. This finding was consistent in both our sensitivity analysis restricting the sample to low-educated women (more likely to be eligible for BFP) and in our additional race-specific models with BFP as a covariate. This could be explained because BFP recipients are poorer than BFP non-recipients (since low income is a criterion to receive BFP) and lower income is also a risk factor for CC mortality. However, our education adjustments might not have been able to completely account for income differences between BFP recipients vs. non-recipients. Moreover, our exposure of interest is race and not BFP, and thus examination of the BFP-mortality association would probably have required consideration of a different set of confounders. The effect of BFP on CC mortality deserves further exploration, preferably using impact evaluation methods, with more explicitly causal analyses (e.g., quasi-experimental designs using inverse probability weighting to receive BFP, among BFP-eligible women only).

An important limitation of our study is the observational design, limiting causal conclusions. We lack data on income, which would be more adequate to identify those women eligible to receive BFP. However, education is highly correlated to income in Brazil^32^ and was used instead. Despite this, a potential bias due to unmeasured confounding by income or selection into BFP cannot be disregarded. We also lack data on access to health care and clinical variables (e.g., Pap test utilization, HPV vaccination, CC stage at diagnosis and treatment). However, according to our conceptual model (Fig. 1), access to health care represents a potential mediator in the race-BFP-mortality relationship. Thus, adjusting for it would not be indicated. Lastly, the 100MCohort represents the poorest 55% of Brazil’s population, which limits the generalizability of our findings. However, as wealthier White women are under-represented in our study, one might expect our associations to possibly underestimate true racial inequities and be even larger among the general Brazilian population.

Strengths of the study include the use of real-world linked data, based on reasonably good quality cervical cancer nationwide mortality registries in terms of coverage and completeness. Moreover, sensitivity analyses restricted to municipalities with better-quality mortality data yielded similar findings. In addition, the 100MCohort is uniquely positioned to study the effects of social policies on the health of specific population subgroups (e.g., marginalized racial groups), due to the individual-level data and large sample size.

In conclusion, our study revealed the highest vulnerability to CC mortality among Indigenous women. Although Indigenous people constitute 10% of the Latin American population,^18^ the burden of CC among them remains largely unknown due to lack of data. Reaching health equity requires not only showing evidence on racial differentials in health, but also actions (e.g., policy-level interventions) to eliminate them. Our findings demonstrated the potential of a conditional cash transfer programme (the BFP) in reducing racial inequities in CC mortality, possibly by improving women’s income and access to health services, especially for Black, Parda and Indigenous women. As policy implications and external public health recommendations, we suggest affirmative actions to ensure a more racially diverse health care workforce to reduce racial bias in health care and institutional racism. We also suggest the inclusion of HPV vaccination and CC screening (e.g., Pap testing) to BFP conditionalities, with special attention to Indigenous, Black and Parda women. In Brazil’s huge territory with stark inequalities, feasibility issues (ensuring supply and accessibility of services, especially in remote areas) must also be achieved.

## Contributors

JMNG, MB, EMLA and JMP conceptualized the study. JMNG conducted the analysis and wrote the manuscript. JMNG and JMP have accessed and verified the data. All authors contributed to the interpretation of data for the study, reviewed it critically for important intellectual content, approved the final version of the manuscript, and agreed to be accountable for all aspects of the work in ensuring that questions related to the accuracy or integrity of any part of the work are appropriately investigated and resolved.

## Data sharing statement

All data from this study were obtained from CIDACS (Center for Data and Knowledge Integration for Health). These data contain sensitive information and due to privacy regulations from the Brazilian Ethics Committee, are not openly available. Upon reasonable request, with express permission from CIDACS (cidacs.curadoria@fiocruz.br) and approval from the Ethics Committee, controlled access to the data is possible. Open access (individualized or aggregated) datasets from publicly available sources are offered by CIDACS at a de-identified data platform (PDD) (Cidacs » Plataforma de Dados Desidentificados).

## Declaration of interests

PC declares an UK National Institute of Health payment. All other authors declare no competing interests.

## Appendix A Supplementary data

Supplementary Fig. S1 and Tables S1–S6

Membership of SEDHI
