# Supplementary material for: Racial inequities in cervical cancer mortality and the role of the Bolsa Família conditional cash transfer programme: results from the 100 Million Brazilian Cohort
Source: Lancet Reg Health Am. 2026 Jul 14;62:101562. doi: 10.1016/j.lana.2026.101562 (PMC13382594; doi:10.1016/j.lana.2026.101562)
Supplement: Supplementary Fig. S1 and Tables S1–S6 [file mmc1.pdf]

Table of contents - Supplementary material

Supplementary Table 1. Characteristics of the study population, by Bolsa Família Programme recipient. 100 Million Brazilian Cohort (2004-2015), N=18,291,600 women aged 18-100 years.

Supplementary Figure 1. Cervical cancer age-standardized* mortality rates per 100,000 women-years, by race and Bolsa Família recipient groups (Yes/No). 100 Million Brazilian Cohort (2004-2015), N=18,291,600 women aged 18-100 years.

Supplementary Table 2. Mortality rate ratios and 95%CIs for the association between race and cervical cancer mortality obtained from sensitivity analysis, restricting the sample women from Brazilian municipalities known to have high proportion of death registration (≥95%). 100 Million Brazilian Cohort (2004-2015), N=7,245,354.

Supplementary Table 3. Mortality rate ratios and 95%CI for the association between race and cervical cancer mortality, obtained from sensitivity analysis restricting the sample to low-educated women (more likely to be eligible for the BFP). 100 Million Brazilian Cohort (2004-2015), N=8,059,473 women.

Supplementary Table 4. Mortality rate ratios and 95%CI for the association between race and cervical cancer mortality, additionally adjusted for Brazilian region of residence. 100 Million Brazilian Cohort (2004-2015), N=18,289,411 women.

Supplementary Table 5. Association of BFP recipient with cervical cancer mortality, stratified by race. 100 Million Brazilian Cohort (2004-2015), N=18,291,600 women aged 18-100y.

Supplementary Table 6. Mortality rate ratios and 95%CI for the association between race and cervical cancer mortality, obtained from quasi-poisson model. 100 Million Brazilian Cohort (2004-2015), N=18,291,600 women.

Supplementary Table 1. Characteristics of the study population, by Bolsa Família Programme recipient. 100 Million Brazilian Cohort (2004-2015), N=18,291,600 women aged 18-100 years.

	**Bolsa Família recipient**		
Variables	Yes<br>n=13,026,278	No<br>n=5,265,322	*P*-value
Cervical cancer deaths, n (%)	7,468 (0.06)	2,105 (0.04)	< 0.001
Age at baseline, mean (SD)	32.9 (12.5)	47.3 (18.1)	< 0.001
Self-declared race, n (%)			
White	3,984,316 (30.6)	2,349,595 (44.6)	< 0.001
Brown	7,679,654 (59.0)	2,509,686 (47.7)	
Black	1,218,409 (9.3)	363,170 (6.9)	
Asian	54,741 (0.4)	33,728 (0.6)	
Indigenous	89,158 (0.7)	9,143 (0.2)	
Education in years, n (%)			
>9	3,171,513 (24.3)	1,910,272 (36.3)	< 0.001
6-9	4,283,902 (32.9)	866,440 (16.5)	
<=5	5,570,863 (42.8)	2,488,610 (47.3)	
Area of residence, n (%)			
Urban	9,983,252 (76.6)	4,598,908 (87.3)	< 0.001
Rural	3,043,026 (23.4)	666,414 (12.7)	

Supplementary Figure 1. Cervical cancer age-standardized* mortality rates per 100,000 women-years, by race and Bolsa Família recipient groups (Yes/No). 100 Million Brazilian Cohort (2004-2015), N=18,291,600 women aged 18-100 years.

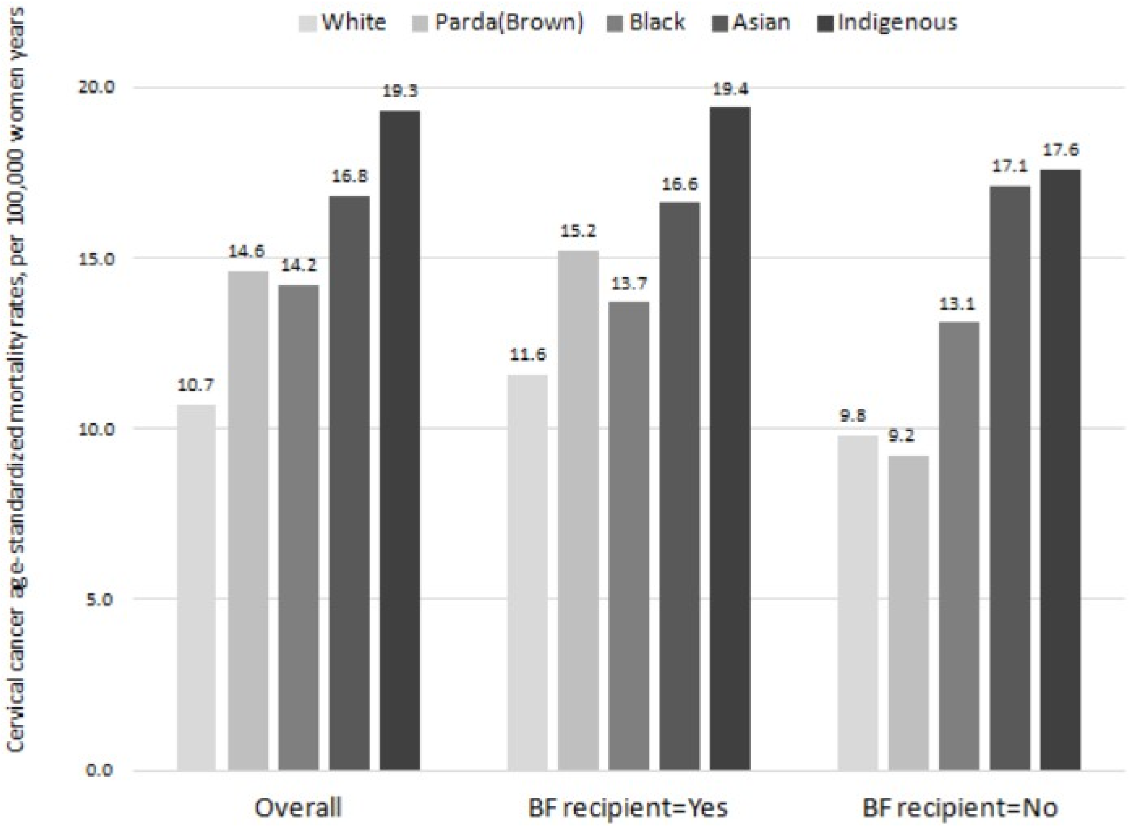

Note: BF, Bolsa Família.

* Rates were age-standardized with 5-year age groups except for ages ≥70 years which were collapsed into a single category, using the Segi's world population.

Supplementary Table 2. Mortality rate ratios and 95%CIs for the association between race and cervical cancer mortality obtained from sensitivity analysis, restricting the sample women from Brazilian municipalities known to have high proportion of death registration (≥95%). 100 Million Brazilian Cohort (2004-2015), N=7,245,354.

	**All municipalities (N=18,291,600)**	**Municipalities with death registration rate ≥95% (N=7,245,354)**
Variables	**MRR (95%CI), Model 4[a]**	**MRR (95%CI), Model 4[a]**
Race		
Parda (Brown) vs White	1.28 (1.22,1.34)[b]	1.21 (1.13,1.30)[b]
Black vs White	1.20 (1.12,1.29)[b]	1.14 (1.02,1.28)[d]
Asian vs White	1.55 (1.16,2.06)[c]	1.34 (0.78,2.31)[e]
Indigenous vs White	1.78 (1.40,2.25)[b]	2.05 (1.33,3.16)[f]
Age at baseline per 5-year increase	1.04 (1.04,1.04)[b]	1.04 (1.03,1.04)[b]
Education		
6-9 years vs >9 years	1.74 (1.59,1.89)[b]	1.80 (1.57,2.06)[b]
<= 5 years vs >9 years	2.29 (2.10,2.49)[b]	2.15 (1.88,2.45)[b]
Area of residence		
Rural vs Urban	0.70 (0.66,0.74)[b]	0.70 (0.62,0.79)[b]
Year of enrolment per 1-year increase	0.97 (0.96,0.98)[b]	0.98 (0.97,0.99)[b]
Bolsa Família Programme recipient		
No vs Yes	0.68 (0.64,0.72)[b]	0.70 (0.64,0.77)[b]

Note: aMRR, adjusted-Mortality rate ratio. CI, Confidence interval.
[a] Model 4: + Age + Education + Area of residence + Year of enrolment + BFP recipient.
[b] P-value<0.001, [c] P-value=0.003, [d] P-value=0.018, [e] P-value=0.295, [f] P-value=0.001

Supplementary Table 3. Mortality rate ratios and 95%CI for the association between race and cervical cancer mortality, obtained from sensitivity analysis restricting the sample to low-educated women (more likely to be eligible for the BFP). 100 Million Brazilian Cohort (2004-2015), N=8,079,479 women.

	**All women (N=18,291,600)**	**Low-educated women only [a] (N=8,059,473)**
Variables	**aMRR (95%CI), Model 4[b]**	**aMRR (95%CI), Model 4[c]**
Race		
Parda (Brown) vs White	1.28 (1.22,1.34)*	1.35 (1.28,1.43)*
Black vs White	1.20 (1.12,1.29)*	1.25 (1.14,1.36)*
Asian vs White	1.55 (1.16,2.06)**	1.34 (0.92,1.94)***
Indigenous vs White	1.78 (1.40,2.25)*	1.92 (1.48,2.48)*
Age at baseline per 5-year increase	1.04 (1.04,1.04)*	1.04 (1.04,1.04)*
Education		
6-9 years vs >9 years	1.74 (1.59,1.89)*	-
<= 5 years vs >9 years	2.29 (2.10,2.49)*	-
Area of residence		
Rural vs Urban	0.70 (0.66,0.74)*	0.70 (0.66,0.75)*
Year of enrolment per 1-year increase	0.97 (0.96,0.98)*	0.97 (0.96,0.98)*
Bolsa Família Programme recipient		
No vs Yes	0.68 (0.64,0.72)*	0.68 (0.63,0.72)*

Note: aMRR, adjusted-Mortality rate ratio. CI, Confidence interval.

[a] Low-educated women were defined as those who never went to school or attended ≤5 years of education

[b] Adjusted for: + Age + Education + Area of residence + Year of enrolment + BF recipient (yes/no)

[c] Adjusted for: + Age + Area of residence + Year of enrolment + BF recipient (yes/no)

* P-value<0.001, ** P-value=0.003, *** P-value=0.128

Supplementary Table 4. Mortality rate ratios and 95%CI for the association between race and cervical cancer mortality, additionally adjusted for Brazilian region of residence. 100 Million Brazilian Cohort (2004-2015), N=18,289,411 women.

	aMRR (95%CI)	
Variables	**Model 4[a]**	**Model 5[b]**
Race		
Parda (Brown) vs White	1.28 (1.22,1.34)*	1.23 (1.17,1.29)*
Black vs White	1.20 (1.12,1.29)*	1.22 (1.14,1.32)*
Asian vs White	1.55 (1.16,2.06)**	1.51 (1.13,2.00)***
Indigenous vs White	1.78 (1.40,2.25)*	1.46 (1.15,1.86)****
Age at baseline per 5-year increase	1.04 (1.04,1.04)*	1.04 (1.04,1.05)*
Education		
6-9 years vs >9 years	1.74 (1.59,1.89)*	1.74 (1.59,1.90)*
<= 5 years vs >9 years	2.29 (2.10,2.49)*	2.28 (2.09,2.48)*
Area of residence		
Rural vs Urban	0.70 (0.66,0.74)*	0.68 (0.64,0.72)*
Year of enrolment per 1-year increase	0.97 (0.96,0.98)*	0.97 (0.96,0.98)*
Bolsa Família Programme recipient		
No vs Yes	0.68 (0.64,0.72)*	0.67 (0.64,0.71)*
Region of residence		
North vs Southeast	-	1.91 (1.79,2.04)*
Central-west vs Southeast	-	1.11 (1.05,1.17)*
Northeast vs Southeast	-	1.32 (1.23,1.42)*
South vs Southeast	-	1.28 (1.19,1.39)*

Note: aMRR, adjusted-Mortality rate ratio. CI, Confidence interval.
[a] Model 4: + Age + Education + Area of residence + Year of enrolment + BF recipient (yes/no)
[b] Model 5: Model 4 + Brazilian region of residence
* P-value<0.001, ** P-value=0.003, *** P-value=0.005, **** P-value=0.002

Supplementary Table 5. Association of BFP recipient with cervical cancer mortality[a], stratified by race. 100 Million Brazilian Cohort (2004-2015), N=18,291,600 women aged 18-100y.

	White	Parda (Brown)	Black	Asian	Indigenous
	n=6,333,911	n=10,189,340	n=1,581,579	n=88,469	n=98,301
Variables	aMRR (95%CI)	aMRR (95%CI)	aMRR (95%CI)	aMRR (95%CI)	aMRR (95%CI)
Bolsa Família Programme recipient					
No vs Yes	0.68 (0.61,0.75)	0.66 (0.61,0.71)	0.83 (0.70,1.00)	0.81 (0.36,1.82)	0.54 (0.22,1.32)
Age at baseline per 5-year increase	1.04 (1.04,1.04)	1.04 (1.04,1.05)	1.04 (1.04,1.05)	1.05 (1.03,1.07)	1.04 (1.03,1.06)
Education					
6-9 years vs >9 years	1.73 (1.48,2.01)	1.73 (1.54,1.95)	1.76 (1.31,2.37)	2.65 (0.86,8.10)	1.01 (0.25,4.07)
<= 5 years vs >9 years	2.10 (1.81,2.44)	2.42 (2.17,2.71)	2.23 (1.67,2.97)	1.60 (0.52,4.98)	2.41 (0.73,7.99)
Area of residence					
Rural vs Urban	0.60 (0.54,0.68)	0.72 (0.67,0.77)	0.74 (0.62,0.89)	0.96 (0.48,1.91)	0.84 (0.51,1.39)
Year of enrolment per 1-year increase	0.97 (0.95,0.98)	0.98 (0.96,0.99)	0.98 (0.95,1.01)	0.91 (0.81,1.01)	1.10 (1.01,1.20)

Note: aMRR, Adjusted-Mortality rate ratio. CI, Confidence interval. BFP, Bolsa Família Programme

[a] Adjusted for age + education + area of residence (Rural/urban) + year of enrolment. P for interaction = 0.017

Supplementary Table 6. Mortality rate ratios and 95%CI for the association between race and cervical cancer mortality, obtained from quasi-Poisson model. 100 Million Brazilian Cohort (2004-2015), N=18,291,600 women.

	Model 4[a]		Model 4[b]	
Variables	**aMRR (95%CI)**	**SEs**	**aMRR (95%CI)**	**SEs**
Race				
Parda (Brown) vs White	1.28 (1.22,1.34)*	0.03	1.30 (1.24,1.36)*	0.03
Black vs White	1.20 (1.12,1.29)*	0.04	1.20 (1.12,1.29)*	0.04
Asian vs White	1.55 (1.16,2.06)**	0.22	1.53 (1.15,2.04)**	0.22
Indigenous vs White	1.78 (1.40,2.25)*	0.22	1.80 (1.41,2.28)*	0.22
Age at baseline per 5-year increase	1.04 (1.04,1.04)*	0.001	1.04 (1.04,1.04)*	0.001
Education				
6-9 years vs >9 years	1.74 (1.59,1.89)*	0.08	1.81 (1.66,1.98)*	0.08
<= 5 years vs >9 years	2.29 (2.10,2.49)*	0.10	2.39 (2.19,2.60)*	0.10
Area of residence				
Rural vs Urban	0.70 (0.66,0.74)*	0.02	0.70 (0.67,0.74)*	0.02
Year of enrolment per 1-year increase	0.97 (0.96,0.98)*	0.004	0.83 (0.82,0.83)*	0.003
Bolsa Família Program recipient				
No vs Yes	0.68 (0.64,0.72)*	0.02	0.67 (0.63,0.71)*	0.02

Note: aMRR, adjusted-Mortality rate ratio. CI, Confidence interval. SEs, Standard errors.
[a] Model 4: Fully adjusted model, obtained from the standard Poisson model.
[b] Model 4: Fully adjusted model, obtained from a quasi-Poisson model to account for overdispersion.
* P-value<0.001, ** P-value=0.003
