# Supplementary material for: Racial inequities in cervical cancer mortality and the role of the Bolsa Família conditional cash transfer programme: results from the 100 Million Brazilian Cohort
Source: Lancet Reg Health Am. 2026 Jul 14;62:101562. doi: 10.1016/j.lana.2026.101562 (PMC13382594; doi:10.1016/j.lana.2026.101562)
Supplement: Membership of SEDHI [file mmc2.docx]

**Membership of the Unit on the Social and Environmental Determinants of Health Inequalities (SEDHI)**

Alvarez, Pablo, Universidad Internacional del Ecuador, Ecuador ([palvarez@uide.edu.ec](mailto:palvarez@uide.edu.ec))

Almeida, Cristiana, Fiocruz, Brazil ([cristiana.almeida@fiocruz.br](mailto:cristiana.almeida@fiocruz.br))

Aldaz Barreno, Cristina Elizabeth, Universidad Internacional del Ecuador, Ecuador ([craldazba@uide.edu.ec](mailto:craldazba@uide.edu.ec))

Andrade, Roberto F.S., Physics Institute and Centre for Data and Knowledge Integration for Health-CIDACS, Federal University of Bahia/Fiocruz, Salvador, Brazil ([randrade@ufba.br](mailto:randrade@ufba.br))

Andrade Ortiz, Diego Oswaldo,  Universidad Internacional del Ecuador, Quito , Ecuador ([diandradeor@uide.edu.ec](mailto:diandradeor@uide.edu.ec))

Anjos, EF, National School of Public Health (ENSP), Centre for Data and Knowledge Integration for Health (CIDACS), Fiocruz, Brazil ([eduarda.anjos@fiocruz.br](mailto:eduarda.anjos@fiocruz.br))

Araújo da Cruz Casais e Silva, Luiz Gustavo, Fiocruz, Brazil ([lgcasais3@gmail.com](mailto:lgcasais3@gmail.com))

Arcos Garcia, Pamela Alejandra, Universidad Internacional del Ecuador, Ecuador ([parcos@uide.edu.ec](mailto:parcos@uide.edu.ec))

Barreto, Mauricio L, Centre for Data and Knowledge Integration for Health-CIDACS, Fiocruz, Salvador, Brazil ([mauricio.barreto@fiocruz.br](mailto:mauricio.barreto@fiocruz.br))

Bernal RTI, Programa de Pós-graduação em Saúde Pública, Universidade Federal de Minas Gerais, Belo Horizonte, Brazil ([reginabernal@terra.com.br](mailto:reginabernal@terra.com.br))

Borbor Cordova, Mercy Julia, Escuela Superior Politécnica del Litoral, Ecuador ([meborbor@espol.edu.ec](mailto:meborbor@espol.edu.ec))

Brickley, Elizabeth, London School of Hygiene & Tropical Medicine, UK ([elizabeth.brickley@lshtm.ac.uk](mailto:elizabeth.brickley@lshtm.ac.uk))

Burke, Claire, University of Glasgow, Glasgow, UK ([claire.burke@glasgow.ac.uk](mailto:claire.burke@glasgow.ac.uk))

Butler, Brenda, University of Glasgow, Glasgow, UK ([brenda.butler@glasgow.ac.uk](mailto:brenda.butler@glasgow.ac.uk))

Calderon Huachi, Nina Micaela, University of Glasgow, Glasgow, UK ([2947863C@student.gla.ac.uk](mailto:2947863C@student.gla.ac.uk))

Campbell, Desmond D, School of Health and Wellbeing, University of Glasgow, Glasgow, UK ([desmond.campbell@glasgow.ac.uk](mailto:desmond.campbell@glasgow.ac.uk))

Campbell, Mhairi, University of Glasgow, UK ([mhairi.campbell@glasgow.ac.uk](mailto:mhairi.campbell@glasgow.ac.uk))

Carrero, Roberto, Fiocruz, Brazil ([roberto@perezcarreiro.org](mailto:roberto@perezcarreiro.org))

Cardoso Laís SM, Programa de Pós-graduação em Saúde Pública, Universidade Federal de Minas Gerais, Belo Horizonte, Brazil ([laissmcardoso@gmail.com](mailto:laissmcardoso@gmail.com))

Cerezo, Jose L, University of Glasgow, Glasgow, UK ([jose-luis.cerezo-Zambudio@glasgow.ac.uk](mailto:jose-luis.cerezo-Zambudio@glasgow.ac.uk))

Chis Ster, Irina, St George’s University of London, London, UK ([ichisste@sgul.ac.uk](mailto:ichisste@sgul.ac.uk))

Constatine Tigua, Annabel del Rocio, Escuela Superior Politécnica del Litoral, , Ecuador ([anndcons@espol.edu.ec](mailto:anndcons@espol.edu.ec))

Cooper, Philip J, Institute of Infection and Immunity, St George’s University of London, London, UK ([pcooper@sgul.ac.uk](mailto:pcooper@sgul.ac.uk))

Costa, Marianne, Fiocruz, Salvador, Brazil ([marianne.lage@fiocruz.br](mailto:marianne.lage@fiocruz.br))

Correa Matta, Gustavo, Fiocruz, Brazil ([gcmatta@gmail.com](mailto:gcmatta@gmail.com))

Craig , Peter, University of Glasgow, Glasgow, UK ([peter.craig@glasgow.ac.uk](mailto:peter.craig@glasgow.ac.uk))

Cruz, Enny Paixao, London School of Hygiene & Tropical Medicine, ([enny.cruz@lshtm.ac.uk](mailto:enny.cruz@lshtm.ac.uk))

de Araujo Almeida, Bethânia, Fiocruz, Brazil ([bethania.almeida@fiocruz.br](mailto:bethania.almeida@fiocruz.br))

de Carvalho Neto, Edgar Marcelino, Fiocruz, Brazil ([edgar.neto@fiocruz.br](mailto:edgar.neto@fiocruz.br))

de Jesus Neves, Felix, Fiocruz, Brazil ([felixnevesjr@hotmail.com](mailto:felixnevesjr@hotmail.com))

de Oliveira Ramos, Dandara, Fiocruz, Brazil ([dandara.ramos@ufba.br](mailto:dandara.ramos@ufba.br))

de Souza Dias, Francine, Fiocruz, Brazil ([francine.dias@fiocruz.br](mailto:francine.dias@fiocruz.br))

Dundas, Ruth, MRC/CSO Social and Public Health Sciences Unit, University of Glasgow, Glasgow, UK ([ruth.dundas@glasgow.ac.uk](mailto:ruth.dundas@glasgow.ac.uk))

Einloft, ABN, Centre for Data and Knowledge Integration for Health-CIDACS, Fiocruz, Brazil ([ariadne.einloft@fiocruz.br](mailto:ariadne.einloft@fiocruz.br))

Emanuel da Silva, Lucas, Federal University of Bahia, Brazil ([lucasemanuel@ufba.br](mailto:lucasemanuel@ufba.br))

Fonseca, A. A., Fiocruz, Salvador, Brazil ([adalton.anjos@gmail.com](mailto:adalton.anjos@gmail.com))

Fernandes Silva Andrade, Roberto, Fiocruz, Brazil ([randrade@ufba.br](mailto:randrade@ufba.br))

Fiaccone Leovigildo, Rosemeire, Federal University of Bahia, Brazil ([rose.fiaccone@gmail.com](mailto:rose.fiaccone@gmail.com))

Flores-Quispe, María del Pilar, Fiocruz, Brazil ([maria.quispe@fiocruz.br](mailto:maria.quispe@fiocruz.br))

Franco Sansigolo Kerr, Ligia, Federal University of Ceará, Fortaleza, CE, Brazil ([ligiakerr@gmail.com](mailto:ligiakerr@gmail.com))

Ferreira dos Santos, Gervásio, Federal University of Bahia, Brazil ([gervasios@ufba.br](mailto:gervasios@ufba.br))

Goes, Emmanuelle, Fiocruz, Brazil ([emanuellegoes@gmail.com](mailto:emanuellegoes@gmail.com))

Gonzaga, Marcos, University of Rio Grande do Norte (UFRN), Brazil ([marcos.gonzaga@ufrn.br](mailto:marcos.gonzaga@ufrn.br))

Gualan, Monsermin, Universidad Internacional del Ecuador, Ecuador ([mogualanch@uide.edu.ec](mailto:mogualanch@uide.edu.ec))

Guimarães, JMN, Center for Data and Knowledge Integration for Health-CIDACS, Fiocruz, Salvador, Brazil ([joannaguimaraes@hotmail.com](mailto:joannaguimaraes@hotmail.com))

Hargreaves, Sally, Institute for Infection and Immunity, St George’s University of London, London, UK ([s.hargreaves@sgul.ac.uk](mailto:s.hargreaves@sgul.ac.uk))

Harron, Katie , Population, Policy and Practice, Great Ormond Street Institute of Child Health, University College London, London, UK ([k.harron@ucl.ac.uk](mailto:k.harron@ucl.ac.uk))

Herculano de Morais, Évelin Angélica, Universidad Federal de Minas Gerais, Brazil ([moraisevelinah@gmail.com](mailto:moraisevelinah@gmail.com))

Ichihara, Maria Yury, Cidacs, Fiocruz, Salvador, BA, Brazil ([maria.yury@fiocruz.br](mailto:maria.yury@fiocruz.br))

Katikireddi, Vittal, University of Glasgow, Glasgow, UK ([vittal.katikireddi@glasgow.ac.uk](mailto:vittal.katikireddi@glasgow.ac.uk))

Kendall, Carl, Graduate Program in Public Health, Federal University of Ceará , Fortaleza CE, Brazil ([carl.kendall@gmail.com](mailto:carl.kendall@gmail.com))

Leyland, AH, MRC/CSO Social and Public Health Sciences Unit, University of Glasgow, Glasgow, UK ([alastair.leyland@glasgow.ac.uk](mailto:alastair.leyland@glasgow.ac.uk))

Lilford, RJ, Institute of Applied Health, University of Birmingham, Birmingham, UK ([r.j.lilford@bham.ac.uk](mailto:r.j.lilford@bham.ac.uk))

Llangari Arizo, Luz Marina, Universidad Internacional del Ecuador, Ecuador ([lullangari@uide.edu.ec](mailto:lullangari@uide.edu.ec))

Lopes, David, Center for Data and Knowledge Integration for Health-CIDACS, Fiocruz, Salvador, BA, Brazil ([davidlopes.educacao@gmail.com](mailto:davidlopes.educacao@gmail.com))

Lowe, Rachel, London School of Hygiene & Tropical Medicine, UK ([rachel.lowe@lshtm.ac.uk](mailto:rachel.lowe@lshtm.ac.uk))

Lugo, Daniela, Universidad Internacional del Ecuador, Ecuador ([dalugoro@uide.edu.ec](mailto:dalugoro@uide.edu.ec))

Macdonald, Sara, University of Glasgow, UK ([sara.macdonald@glasgow.ac.uk](mailto:sara.macdonald@glasgow.ac.uk))

Malta DC, Departamento de Enfermagem Materno-Infantil e Saúde Pública, Universidade Federal de Minas Gerais, Belo Horizonte, Brazil ([dcmalta@uol.com.br](mailto:dcmalta@uol.com.br))

Mendes Pereira, Adelyne, Fiocruz, Brazil ([adelyne.mendes@fiocruz.br](mailto:adelyne.mendes@fiocruz.br))

Mendoza Ruiz, Adriana, Fiocruz, , Brazil ([adriana.mendoza@fiocruz.br](mailto:adriana.mendoza@fiocruz.br))

Moncayo Benalcazar, Ana Lucía, Centre for Research on Health in Latin America, ([amoncayo708@puce.edu.ec](mailto:amoncayo708@puce.edu.ec))

Moraes Pimenta, Denise, Fiocruz, Sao Paulo, Brazil ([denise.mpimenta@fiocruz.br](mailto:denise.mpimenta@fiocruz.br))

Navarrete Chavez, Grace, Universidad Internacional del Ecuador, Quito/Glasgow, UK ([2947619N@student.gla.ac.uk](mailto:2947619N@student.gla.ac.uk))

Nascimento, Erica Miranda, Fiotec, Brazil ([ericanascimento@fiotec.fiocruz.br](mailto:ericanascimento@fiotec.fiocruz.br))

Nunes de Carvalho, Rumão Batista, Brazil ([rumao.carvalho@fiocruz.br](mailto:rumao.carvalho@fiocruz.br))

Oliveira Junker, Sara Caroline, Fiotec, Brazil ([sarajunker@fiotec.fiocruz.br](mailto:sarajunker@fiotec.fiocruz.br))

Oliveira Lima Sena, Samila, Fiocruz, Brazil ([samila.sena@fiocruz.br](mailto:samila.sena@fiocruz.br))

Jonathan R Olsen, MRC/CSO Social and Public Health Sciences Unit, University of Glasgow, Glasgow, UK ([jonathan.olsen@glasgow.ac.uk](mailto:jonathan.olsen@glasgow.ac.uk))

Oliveira, Suelen, National School of Public Health (ENSP), Fiocruz, Rio de Janeiro, Brazil  ([suelen.coliveira@fiocruz.br](mailto:suelen.coliveira@fiocruz.br))

Ortelan, Naiá, Fiocruz, Brazil ([naia.ortelan@fiocruz.br](mailto:naia.ortelan@fiocruz.br))

Pinto Junior, EP, Center for Data and Knowledge Integration for Health-CIDACS, Fiocruz, Salvador, Brazil ([elzo.junior@fiocruz.br](mailto:elzo.junior@fiocruz.br))

Pita, Robespierre, Fiocruz, Brazil ([pierre.pita@gmail.com](mailto:pierre.pita@gmail.com))

Julia M Pescarini, Centro de Integração de Dados e Conhecimentos para Saude (CIDACS), Instituto Gongalo Moniz, Fiocruz, Salvador, Brazil ([Julia.Pescarini1@lshtm.ac.uk](mailto:Julia.Pescarini1@lshtm.ac.uk))

Ribeiro, Rita, Federal University of Bahia, Brazil ([ritaribeiroufba@gmail.com](mailto:ritaribeiroufba@gmail.com))

Rodriguez Alvarado, Rodrigo Alejandro, Universidad Internacional del Ecuador, Ecuador ([rorodriguezal@uide.edu.ec](mailto:rorodriguezal@uide.edu.ec))

Batul Rojeab Bravo, St George’s University of London, London, UK ([m2307408@sgul.ac.uk](mailto:m2307408@sgul.ac.uk))

Romero Sandoval, Natalia Cristina, Universidad Internacional del Ecuador, Quito, Ecuador ([nromero@uide.edu.ec](mailto:nromero@uide.edu.ec))

Revoredo, Fernanda, Fiocruz, Salvador, Brazil ([fernanda.revoredo@fiocruz.br](mailto:fernanda.revoredo@fiocruz.br))

Ruano, Maria Alejandra, Facultad de Ciencias Sociales y Humanísticas, Escuela Superior Politecnica del Litoral, Guayaquil, Ecuador ([maruano@espol.edu.ec](mailto:maruano@espol.edu.ec))

Rui Chng, Nai, University of Glasgow, UK ([NaiRui.Chng@glasgow.ac.uk](mailto:NaiRui.Chng@glasgow.ac.uk))

Ruiz, Ronal, Universidad Internacional del Ecuador, Ecuador ([roruiz@uide.edu.ec](mailto:roruiz@uide.edu.ec))

Sande, Raphael, Fiocruz, Brazil ([raphael.sande@fiocruz.br](mailto:raphael.sande@fiocruz.br))

Santos de Jesus, T., Centre for Data and Knowledge Integration for Health-CIDACS, Fiocruz, Salvador, Brazil ([talita.jesus@fiocruz.br](mailto:talita.jesus@fiocruz.br))

Santucci, Paula M, Centre for Data and Knowledge Integration for Health-CIDACS, Fiocruz, Salvador, BA, Brazil ([paulamsantucci@gmail.com](mailto:paulamsantucci@gmail.com))

Scandrett, Katie Ellen, University of Birmingham, UK ([k.e.scandrett@bham.ac.uk](mailto:k.e.scandrett@bham.ac.uk))

Sebastiao, Mariana, Fiocruz, Brazil ([mariana.sebastiao@fiocruz.br](mailto:mariana.sebastiao@fiocruz.br))

Silva de Jesus, Viviane, Fiocruz, Brazil ([vivi_humaniza@hotmail.com](mailto:vivi_humaniza@hotmail.com))

Shimonovich, Michal, University of Glasgow, Glasgow, UK ([michal.shimonovich@glasgow.ac.uk](mailto:michal.shimonovich@glasgow.ac.uk))

Souza , Maira Lima, Fiocruz, Brazil ([maira.souza@fiocruz.br](mailto:maira.souza@fiocruz.br))

Sousa Filho, J.F., Fiocruz, Brazil ([jose.ffilho@fiocruz.br](mailto:jose.ffilho@fiocruz.br))

Tourinho Lima, Raiza, Fiocruz, Brazil ([raizatourinho@gmail.com](mailto:raizatourinho@gmail.com))

Tavares de Souza Junior, Cleônidas, Fiocruz, Brazil ([cleonidas@gmail.com](mailto:cleonidas@gmail.com))

Teles , Carlos, Fiocruz, Brazil ([carlosateles@yahoo.com.br](mailto:carlosateles@yahoo.com.br))

Vera Alcivar, David Gonzalo, Universidad Internacional del Ecuador, Ecuador ([daveraal@uide.edu.ec](mailto:daveraal@uide.edu.ec))

Vieira Machado, Cristiani, Fiocruz, Brazil ([cristiani.machado@fiocruz.br](mailto:cristiani.machado@fiocruz.br))

Wells, Valerie, University of Glasgow, UK ([valerie.wells@glasgow.ac.uk](mailto:valerie.wells@glasgow.ac.uk))

Zurita, Daniel, Universidad Internacional del Ecuador, Ecuador ([dzurita@uide.edu.ec](mailto:dzurita@uide.edu.ec))
